# Increasing Interoperability between Digital Twin Standards and Specifications: Transformation of DTDL to AAS

**DOI:** 10.3390/s23187742

**Published:** 2023-09-07

**Authors:** Carlos Schmidt, Friedrich Volz, Ljiljana Stojanovic, Gerhard Sutschet

**Affiliations:** Fraunhofer IOSB, 76131 Karlsruhe, Germany; carlos.schmidt@iosb.fraunhofer.de (C.S.); ljiljana.stojanovic@iosb.fraunhofer.de (L.S.); gerhard.sutschet@iosb.fraunhofer.de (G.S.)

**Keywords:** Industry 4.0, interoperability, digital twin, asset administration shell, semantic transformation, standards

## Abstract

Although standards and specifications for digital twins aim to create interoperability in Industry 4.0, each standard has its own goals, focuses and representations for digital twins. This paper examines an approach to increasing interoperability between established digital twin specifications by transformation. Accordingly, several specifications are presented and requirements for transformation are examined. Following the feasibility analysis, a mapping between the Digital Twin Definition Language (DTDL) and Asset Administration Shell (AAS) was created. To examine the feasibility of this approach, the transformation was implemented and tested for a physical asset. This paper demonstrates that a generic mapping between DTDL and AAS can be applied for transformation in use cases where DTDL models are provided while AAS is required.

## 1. Introduction

In recent years, several standards and specifications for digital twins have been developed by various organizations and companies with differing goals, focuses and representations for digital twins. Consequently, these existing standards are incompatible with each other, i.e., they do not have the same (i) syntax, (ii) mechanisms for representing properties and behavior, (iii) communication mechanisms and languages or (iv) semantics of properties and behavior. Whilst interoperability of digital twins is one essential feature of digital twin technology, this is only possible if compatible communication mechanisms are used and if the semantics of parameters and behavior are clear to all participating digital twins.

Interoperability mechanisms among digital twins are typically proprietary within the standards. However, as it cannot be expected that in the future there will be only one dominant international standard used to implement digital twins, interoperability between digital twins implemented using different standards will play a major role in digital twin technology in the future.

There are several ways to accomplish interoperability among digital twins within different standards. Firstly, it is important to realize a clear semantics of properties and behavior of the digital twins which can be accomplished either by using the representation for semantics of one of the specifications/standards involved or using a third-party representation, e.g., a common ontology. Secondly, a common communication mechanism must be implemented which can either be achieved by using the already existing mechanism of one standard or again using a third-party mechanism. The latter typically is the costliest option, given that the implementations must be developed for each participating specification/standard.

A simple way to handle these interoperability issues is to transform different standards into each other wherever possible. Various approaches to transformation have been elaborated in the literature; for instance, in addition to a general transformation approach, there are also specific transformations of two standards and Asset Administration Shell (AAS) [1,2,3,4].

The main contributions of this paper are two-fold: First, the models of current relevant standards and specifications are structurally examined and transformation possibilities into the AAS model are investigated. The goal is to enable the transformation of two previously incompatible standards through the definition of a transformation concept. The next step is the development of a suitable software implementation of this concept by means of a bidirectional mapping regarding the model elements of a standard/specification to the respective counterpart in the AAS.

This paper is organized as follows: In Section 2, a list of requirements is presented as well as an example, which is used for the analysis. These requirements are then used in Section 3 to analyze five frequently used standards and specifications for digital twins. Section 4 provides a feasibility analysis of a transformation of the Digital Twins Definition Language (DTDL) into AAS. The metamodel elements of the DTDL are classified in terms of their similarity with the AAS elements in Section 5. Finally, Section 6 presents an implementation for the transformation of the DTDL into AAS.

## 2. Approach

In this section, the requirements for the analysis of the different digital twin specifications/standards are presented. Since the primary concern is the substitutability of standards and specifications with the aim of transforming the models between them, and not with their concrete implementation, aspects such as communication protocols or security aspects of implementations are not considered.

The following general characteristics of the standards and specifications are analyzed for an overview of the standards:

- Version: specifies the version that is analyzed in this paper;
- Context: promotes understanding of the design decisions within the standard or specification;
- Implementations: describes relevance and widespread use of a standard or a specification;
- Serializability: describes information about the portability of models.

For the next step of analyzing the metamodel of the standards and specifications, the following points of analysis are used:

- Structure of the metamodel: names the core elements and their functionality to provide some basic understanding;
- Representation of data: considers the complexity of the constructs, e.g., if complex constructs are possible or only simple datatypes;
- Semantic annotation of data: considers interpretation of data by humans and machine [5];
- Identification of model elements: considers which elements can be uniquely identified and how this is carried out.

Following the analysis of the metamodels, the candidates were tested with respect to the requirements. Moyne et al. [6] derived a list of requirements from an analysis of DT definitions, some of which are also relevant for standardization and specification. These requirements are commonly cited in the domain of DT design principles and frameworks. Table 1 lists some of the relevant comparison requirements that were used to classify and compare the standards and specifications.

**Figure 1:**
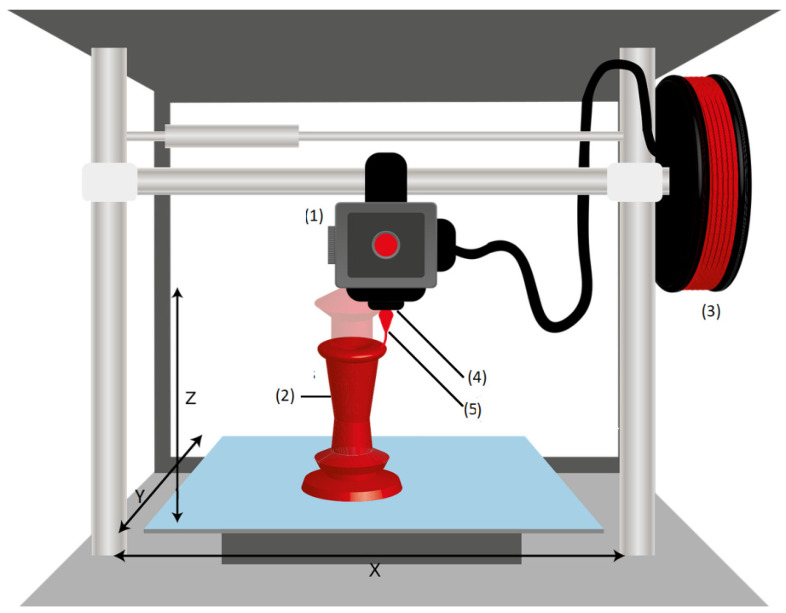
Schematic representation of the 3D printer [7]: (1) print head; (2) element to be printed; (3) proximity sensor; (4) temperature sensor; (5) filament store.

To perform the analysis, a common example is used. It is modeled with each specification and the results are presented in a serialized form. The example is a fictitious asset, namely a 3D printer. In Figure 1, a schematic representation of the 3D printer is shown. The 3D printer (also “asset”) is equipped with some functionalities and properties, which are to be represented by a digital twin. Firstly, manufacturer-specific information about the asset is stored, such as the dimensions of the 3D printer or a manufacturer’s specification. The asset has two sensors (cf. 4 and 5 in Figure 1):

- A proximity sensor, which determines the distance between the print head and the surface;
- A temperature sensor, which is used to measure the temperature of the print head.

An event is triggered as soon as a certain temperature is reached. In addition, the asset has an operation that starts a print program if activated. The name of the program can be specified as an argument, and the return value of the program can be also specified. Finally, the filament store of the 3D printer is modeled as its own digital twin and represents a component of the asset (cf. 3 in Figure 1). The details of the digital twin of the filament storage system will not be discussed further; it shall be considered a “black box”.

## 3. Analysis of the Requirements

In this section, five frequently used standards and specifications for digital twins with respect to the requirements (Table 1) are analyzed. The Asset Administration Shell is presented first, since it is the chosen specification for this work, and interoperability between it and another candidate is to be established. Thus, it should also become clear which properties other standards/specifications should possess in order to be considered for transformation.

### 3.1. Fulfilment of the Requirements by the AAS

The Asset Administration Shell (AAS) was developed by Plattform Industry 4.0 as part of the Industry 4.0 reference architecture model. The standard is divided into three specification parts—the first part dealing with the metamodel and serialization of an AAS, and the second and third parts detailing the AAS communication at runtime and the infrastructure for provisioning AAS instances to each other, respectively [1]. The first part of the specification series provides the relevant information about the metamodel that is vital for a transformation. The current version 2.0.1 of the first part was released in November 2019. Furthermore, version 3.0RC01 is already available as a release candidate [8]. An asset is described by the Industry 4.0 platform as “everything that provides a ‘connection’ for an Industry 4.0 solution”. Additionally, the AAS is described as “implementation of the digital twin”. Table 2 demonstrates how the AAS specification fulfils the requirements for digital twins introduced in Table 1. Point 7 in Table 2 shows that the basic functionality according to [5] is given. This functionality should also be available in the source/target model to guarantee a lossless transformation. One difficulty in the transformation could be the representation of sub-twins. These would have to be modeled as separate AASs and then referenced by the top-level AAS.

### 3.2. Fulfilment of the Requirements by the DTDL

The Digital Twins Definition Language (DTDL) was developed by Microsoft for the Microsoft Azure platform. The specification of the DTDL is available at [9], and it defines the structure and design of the model components, as well as identification and semantics of the data of a digital twin in DTDL format. DTDL is currently in its second version, which was published in June 2020. The specification is defined as “a language, to describe models of IoT plug and play devices, digital twins of devices, and logical digital twins” [9]. Table 3 demonstrates how DTDL fulfills the requirements for digital twins.

An initial work on transformation of AAS to DTDL is given in [3]. The elements of the AAS metamodel are modeled as DTDL elements which exemplifies that a transformation of AAS into DTDL is already possible. However, a transformation in the other direction, i.e., from DTDL models into the AAS metamodel does not yet exist. The basic functionalities according to [5] are given in DTDL. It should be noted that in the DTDL implementation, “Azure Digital Twins” (ADT) functions (commands) are not supported as modeling elements. Consequently, during transformation, some information of an AAS could be lost if the target application is an ADT. A possible facilitator when testing a transformation is the DTDL validator, which detects syntactic errors of a DTDL model.

### 3.3. Fulfilment of the Requirements by the NGSI-LD

The Next Generation Services Interface-Linked Data (NGSI-LD) standard was published by the Context Information Management (CIM) of the ETSI Industry Specification Group (ISG) [10]. It is based on NGSI 9 and 10 from the Open Mobile Alliance (OMA) and NGSIv2 from FIWARE [5]. The current version 1.5.1 was released in November 2021. Some changes to the version discussed by [5] (1.2.2) are the support for Internationalized Resource Identifiers (IRI) and different languages (LanguageProperty). All changes since version 1.2.10 can be found in [10]. The standard is summarized in [10] as follows: “NGSI-LD enables users to provide, consume, and manage context information in a variety of scenarios and involving multiple actors. Context information is modeled as attributes (properties and relationships) of context entities, also known as ‘digital twins’, that represent real-world values”. The NGSI-LD standard is based on the JSON-LD format, and an NGSI-LD element is “any JSON element, defined by the NGSI-LD API” [10]. The fulfillment of the requirements for digital twins is shown in Table 4. The advantages of NGSI-LD include serialization to the JSON-LD format and support for semantically annotated and geographic data. Elements with historical records of their state are currently not supported by the AAS and could present an obstacle in a transformation. In addition, operations cannot be modeled in NGSI-LD which, according to [5], belongs to the basic functionalities of a digital twin.

### 3.4. Fulfilment of the Requirements by Eclipse Vorto

The Eclipse Vorto specification was developed by the Eclipse Foundation. Vorto is currently in version 1.0, which was released in November 2020. Vortolang is a domain-specific language for modeling Vorto twins. Eclipse Vorto addresses the problem that different Internet of Things (IoT) devices send and receive different types of data. Vorto models are intended to provide a normalized API to IoT devices for easy integration into software solutions. Digital twins are described in the Vortolang documentation as follows: “Digital twins are models of entities in the physical world such as (multi-)sensor devices, smart energy power plants, and other entities that participate in IoT solutions” [11]. Implementations of the specification include Eclipse Ditto or the Bosch IoT Suite. In addition, the Eclipse Foundation provides a list of other implementations. The Vorto metamodel was implemented in the Java programming language. Eclipse Vorto models and components of the model can be published in a public directory (Vorto Repository) and published models/components can be used to build other digital twins. In 2021, this service was discontinued and all Vorto elements published until then were uploaded to [11] (folder models). A special feature of Eclipse Vorto is that program code can be generated directly from a created model. With this code, assets can be directly linked to a Vorto implementation via the model. Examples of code generators can be found at [11]. Table 5 shows the requirement fulfilment of Vorto.

The lack of representation for relationships and compositions of digital twins could present an obstacle in a transformation, as both aspects can be modeled in an AAS. In addition, it can be argued that the discontinuation of the Eclipse Vorto Repository service indicates limited use of the standard. Thus, a transformation with Vorto will not be relevant in this paper.

### 3.5. Fulfilment of the Requirements by the (Web of Things) Thing Description

The standard Web of Things (WoT) Thing Description (TD) was developed by the WoT Working Group of the World Wide Web Consortium (W3C) and has been an official recommendation of the W3C since April 2020 [12]. The official version of the Thing Description (TD) is 1.0. Version 1.1 was published as a draft in November 2020 [13]. Version 1.1 introduces the concept of the Thing Model. With a Thing Model, it is possible to create a template of a digital twin, which, for example, gets by with fewer constraints than an actual twin [13]. This concept is similar to the concept of submodel templates in the AAS. In [14], the TD is described as follows: “A TD describes the metadata and interfaces of a Thing”. A Thing is an abstraction of a physical or virtual entity that enables and participates in interactions with the WoT. The goal here is to enable communication between different devices and applications, by using a minimal vocabulary to describe these things. Thing Description models are described in JSON format and are JSON-LD compatible. The Thing Description is implemented, for example, by the Eclipse Thingweb. In addition, several applications of WoT and TD are listed in [15]. The fulfilment of the requirements for digital twins is shown in Table 6. In [2], a transformation from the TD model to the AAS has already been presented and is considered for the more detailed comparison described in this paper.

## 4. Comparison of the Digital Twin Standards and Specifications with the AAS Specification

In the previous section, several standards and specifications for digital twins were introduced. In this section, they will be compared with the AAS specification. For this purpose, not only technical details of the candidates are used, but also their widespread use and relevance are considered. The result of this section is a candidate that will be used later in this paper for the feasibility analysis and transformation approach. Based on the requirements, the candidates are compared with the AAS. Finally, the candidate that performs best in the comparison is selected.

### 4.1. Technical Details

In the following, some technical functionalities and capabilities of digital twins are listed, their importance, specifically in the context of Industry 4.0, is classified, and an approximate sorting is achieved. Based on the definition of a digital twin underlying this work, digital twins are “virtual representations of resources that organize and manage information” [5]. In order to organize and manage information of a resource, this information has to be recorded earlier. This is conducted by telemetry data modeling and is supported by several elements in the AAS, including the property element. In order to guarantee an information flow back to the resource, the so-called functions are important as a model element in the context of Industry 4.0. An AAS could have an Operation submodel element for this purpose. An operation can also have input parameters and return values. Another important capability is the way models and their elements are identified. In order to be uniquely identifiable in both the source model and the target model, both systems should support identifiers that are as similar as possible. This is trivial in AAS, since, in addition to Internationalized Resource Identifier (IRI) and International Registration Data Identifier (IRDI), specifically defined identifiers are possible. To create systems of twins that communicate with each other, references between twins are necessary. Systems of twins are important in the context of Industry 4.0, so that entire production chains can be interlinked via twins. Similarly, compositions of twins are also an important concept for dividing large systems into smaller twins. Such a division is possible with the AAS elements Entity, Relationship and Reference. Furthermore, the way in which data are represented in the twin is generally an important issue. Without semantic annotations, such as units, data cannot be easily interpreted by machines. For this issue, the AAS has conceptual descriptions that allow standardized use of data types. Another capability of digital twins includes events that are sent by the asset. Since events can also be sent by telemetry data, and it is up to the receiver of the data to react to an event, the capability is not considered of fundamental importance in this work. The following functionalities and capabilities are currently not supported by the AAS but provide a holistic view:

- Time-series data can be important for autonomous systems or machines that process data from an asset and make predictions based on temporal relationships. Time-series data are, at the time of writing, not explicitly supported by the AAS [16], but a time-series submodel template will be released in the future.
- Geodata, which cannot be explicitly defined in the AAS, can be emulated by normal properties.
- An additional useful capability of digital twins is the definition of the connection from the digital twin to its physical asset. However, since this connection is not standardized, it is not further considered.
- Since the security aspects of the current AAS specification are only preliminary [8], the definition of security concepts is excluded.

Table 7 compares the AAS with the other candidates. For the purpose of comparison, the order of the capabilities is sorted according to their importance as discussed previously. Cells are only marked with “✓” where the candidate metamodel explicitly defines this functionality, otherwise “X” is used. When the functionality is not yet explicitly defined or realizable with other features, “(✓)” is used. The identifiers are ranked relative to the AAS and are therefore evaluated with respect to the identification possibility, where “0” indicates the reference (AAS). In this regard, the AAS supports the most types of identifiers.

### 4.2. Discussion

In the previous section, the relevance and prevalence of the candidates were discussed by examining efforts around the candidates. Furthermore, technical capabilities and functionalities were sorted according to their importance for AAS to compare the candidates with the AAS (cf. Table 7). The research regarding relevance and diffusion shows a shortcoming of the Eclipse Vorto specification since the public service “Vorto Repository” was discontinued, as well as the integration of the TD standard into Eclipse Ditto. NGSI-LD, on the other hand, demonstrates the use of the standard with several use cases in areas such as Smart City and Smart Heating. Through Microsoft’s development and integration with Azure Digital Twins, DTDL is used by a wide range of companies. Although no commercial use cases for the reference implementation of the TD standard were found in the context of this work, the support in Eclipse Ditto and the transformation into submodels of AAS make TD an interoperable standard that cannot be neglected. The comparison of technical capabilities and functionalities in Table 7 has shown that NGSI-LD is limited in its ability to communicate with the asset due to the lack of functions as a model element. Since Eclipse Vorto does not support relationships and compositions of twins, larger systems cannot be modeled, which is an important element for digital twins in the context of Industry 4.0. DTDL is characterized by the lack of support for events; however, this is compensated for by DTDL telemetry data. The WoT TD performs best in the comparison. Nonetheless, it must be considered that information on connections of a TD to their assets, as well as security definitions, cannot be used directly by AAS implementations. Since Pakala et al. [2] have already proposed a transformation of TD into the AAS and since DTDL transformations only include the AAS → DTDL direction, for this work, DTDL is selected for further feasibility analysis.

## 5. Feasibility Analysis of a Transformation of the DTDL into AAS

In the previous section, the digital twin standards and specifications were compared based on various aspects, the result of which is the selection of the most suitable candidate, DTDL. In this section, the feasibility and the extent of a transformation of DTDL version 2 into AAS version 3.0RC01 will be analyzed. In order to analyze the feasibility of a transformation, approaches by Mayrbäurl et al. [3] and Pakala et al. [2] on transformations between DTDL and AAS or Thing Description (TD) and AAS are examined. In particular, the different approaches are explored to obtain ideas for transformation shown below in this work. Thereafter, the approach for a transformation is defined and a first proposal of a mapping of the elements is shown. For the next step, mapping rules are defined. This is followed by a transformation of the 3D printer example which was previously modeled in DTDL.

### 5.1. Existing Approaches for Transformations

In [4], the authors describe the mapping of a DT description framework to AAS. The approach is based on a smart clamp drilling machine example. In comparison, this paper compares existing DT standards and specifications with the goal of transforming DTDL to AAS.

In [17], the authors presented a solution for mapping DTDL to the OPC UA information model which allows the transformation of each DTDL element into a corresponding OPC UA element. Compared to our transformation, specific mapping rules must be defined, as the AAS metamodel differs from the OPC UA metamodel. Furthermore, the AAS metamodel provides support for many serialization formats, OPC UA being one of them, which further increases the complexity. Finally, an AAS service could provide an HTTP REST endpoint in addition to an OPC UA endpoint, which increases the complexity of the mapping rules.

The lack of interoperability between digital twins of different companies was studied in [1]. To achieve interoperability between digital twins, the authors proposed a flexible approach to transform their information models and applied the proposed solution to a real application scenario in an industrial context by transforming ABB Ability digital twins into AAS. On a conceptual level, the approach in [1] is similar to the approach taken in this paper, as both approaches consider meta-level mapping. Nonetheless, while [1] maps ABB Ability digital twins as the source model, in this paper DTDL models are targeted. Mapping different source metamodels to AAS results in completely different mapping rules and challenges in their implementation.

**Figure 2:**
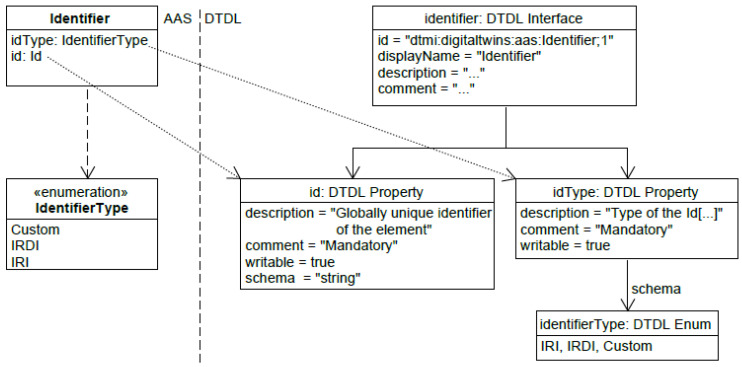
Mapping of the AAS identifier element into the DTDL model.

Mayrbäurl et al. [3] have carried out a transformation of the AAS metamodel into the DTDL metamodel. In this process, each of the elements of the AAS was projected onto DTDL interface elements. Figure 2 provides an example that illustrates the mapping of the AAS identifier element into the DTDL model. The left side shows the identifier of the AAS and the right side shows the mapping into the DTDL model. Thus, the information about the identifier is not lost, although DTDL’s identifier, the Digital Twin Model Identifier, is only a subset of the identifiers supported by the AAS.

In addition, the submodel template “Technical Data” [18] was modeled in DTDL. The four submodel collections that make up the submodel were each projected onto individual DTDL interfaces and their submodel elements were either modeled as DTDL properties or referenced per DTDL component, as shown in Figure 2. For this approach, the AAS elements were projected onto DTDL interface elements. For example, an AAS operation was not mapped to the DTDL equivalent, the command. This approach is most relevant if the target platform of the transformation does not support the Command element, which is the case for Azure Digital Twins.

In [2], the Thing Description of the Web of Things was transformed to AAS. More specifically, a Thing Description model can be imported into an AAS in the form of a submodel. To achieve this, the Thing Description model must be in Javascript Object Notation (JSON) format and the AAS must be used with the AASX Package Manager tool.

The approach is as follows: First, the Thing object is mapped to a submodel. The complex types object, list, array and map of the TD model are grouped into subelement collections (e.g., “properties”) and then mapped onto their own submodel collections. The primitive types string, float, integer and boolean are mapped as AAS qualifiers of the parent element. There are some exceptions to these mapping rules, which can be found in [2]. For example, the Thing identifier was mapped to the submodel identifier.

The use of appropriate AAS elements was avoided (e.g., the operation) for mappings to “avoid a huge hierarchical structure and visual complexity in the representation in the AASX Package Explorer” [2]. Moreover, this avoided “complex mapping rules and enabled an underlying base rule” [2].

### 5.2. Proposed Approach

**Figure 3:**
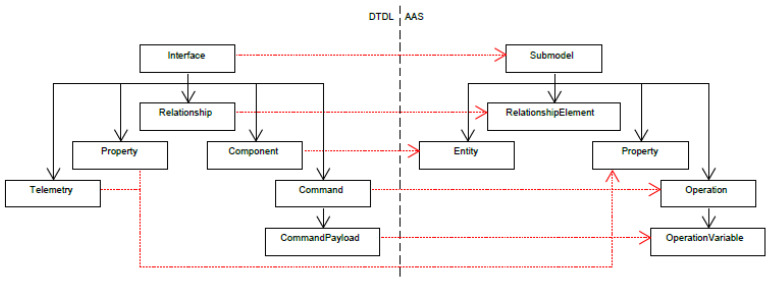
First proposal of a mapping from DTDL to AAS.

The structures of the DTDL and AAS metamodels are primarily considered to determine the approach of the transformation. In addition, the approaches of the previous efforts (see previous section) are used to generate a draft. The structure of the DTDL metamodel is similar to that of the TD metamodel in that the functionality is encapsulated in an element. The elements within the parent element are not further grouped in the DTDL metamodel but are represented in a flat hierarchy. In the TD metamodel, the properties, operations and events are grouped. Due to the similarity in structure, the approach of Pakala et al. [2] is adapted in this work. The superordinate element of the DTDL metamodel interface is mapped to the submodel of the AAS. In addition, the elements contained in the interface are mapped directly to submodel elements within the submodel. A schematic illustration can be found in Figure 3.

Additionally, Figure 3 already contains an initial proposal for mapping the DTDL elements to AAS counterparts. Here both the DTDL telemetry and the DTDL property were assigned to the AAS property. The DTDL property was assigned to the AAS property because the property of the AAS was defined abstractly enough to be suitable for both configuration properties and telemetry data. Fields in AAS elements, which are not a target of a mapping, shall not be set in a transformation unless they have been marked as required in the metamodel. Otherwise, default values should be used to not restrict the respective element.

## 6. Classification of Elements and Definition of Mapping Rules

In this section, the metamodel elements of the DTDL are classified in terms of their similarity. The proposal of a mapping for the elements in the last section (see Figure 3) is considered in more detail. Furthermore, it defines how the identifiers are mapped to DTDL models. In a mapping, elements that occur in the AAS but not in the DTDL are to be neglected, since a mapping of a smaller set into a larger one does not result in a loss of information. The DTDL interface is considered separately as a superordinate element of a model and the remaining DTDL elements are divided into data elements and other elements. The data elements include Telemetry, Property and CommandPayload. The remaining elements are Command, Relationship and Component.

### 6.1. Identification

The DTDL interface is mapped to the submodel of the AAS. The submodel is a so-called identifiable in the AAS hierarchy as a result of its identification by a global identifier, namely the Digital Twin Modeling Identifier (DTMI) of the interface. The remaining DTDL elements are mapped to Referables. Referables are uniquely identified by their idShort within an identifiable and do not have a global identifier. Because this idShort should be unique within a submodel, a mapping of the DTDL field “name” to the idShort field is performed. According to [9], this field is “unique for all content of the interface[-element]”. The mapping of the name field to an idShort is as follows: an idShort always starts with a letter, has at least two characters, and consists of both upper- and lower-case letters, digits from zero to nine, and underscores. A name also starts with a letter and consists of upper- and lower-case letters, numbers from zero to nine and underscores. However, a name can consist of only one letter, so another character must be added when mapping to an idShort. This character is defined as an underscore, which is added to the end of each mapped name. This mapping is injective due to the trivial mapping rule and leads to no conflicts.

### 6.2. DTDL Interface and AAS Submodel

As shown in Table 8, an interface and a submodel possess many similar fields which simplify the interface mapping into a submodel. The identifier of the interface element can be mapped to an AAS identifier by setting the idType to “custom”. Elements, such as a comment or the context of the interface element, which do not have a counterpart, are mapped to generic qualifiers (cf. Section 5.2). The idShort field of the submodel receives a generic identifier, such as “DTDLModel”. Complex data types defined by the schemas field are not mapped at this point but are created within the AAS property elements.

### 6.3. Data Elements

**Figure 4:**
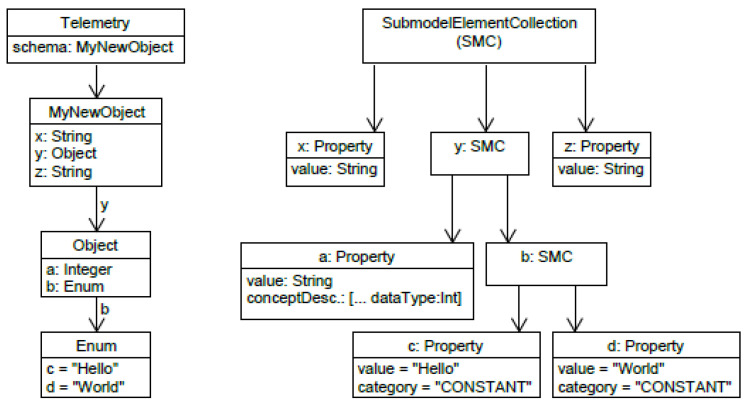
Transformation example of a complex DTDL data type.

The mapping rules of the complex DTDL data types to the data format of the AAS will be discussed in this section. In DTDL models, any complex data type such as an object, array, enumeration or map can be defined. In the AAS data store, a value field is provided, which can be contextualized by units and data type definitions. Primitive data types of the DTDL can be mapped to the value field of the AAS property. Due to the rigid structure, a DTDL object can be mapped to a submodel collection (SubmodelElementCollection). Since the elements of an object also have primitive or complex data types, they are mapped as separate elements within the SubmodelElementCollection. A DTDL enumeration is also a rigid construct (cf. required field enumValues of an enumeration in [9]). As such, SubmodelElementCollection can also be used to map DTDL enumerations. Since the elements of an enumeration can be numbers or strings, they can be modeled by AAS property elements. Figure 4 shows an exemplary transformation of a DTDL complex type.

Due to the variable number of their elements, arrays and maps must be considered separately. While an array with elements of a primitive data type is easily serializable, an array with values of complex data types cannot be serialized in a trivial way. It is possible to map the array to a SubmodelElementCollection and treat the elements separately, as seen with the DTDL object. Given the variable number of array elements at runtime and the fact that the AAS model would have to react to changes in the DTDL model, this approach is not selected. The same issue also exists with DTDL maps because entries can be added at runtime, which would lead to a structural change in the AAS. Finally, DTDL supports geographic data, such as points that can be represented in a coordinate system. These geographical data types are based on arrays with primitive data types and can therefore be mapped to the AAS value in serialized form.

Due to the similarity of the fields of Telemetry, Property and CommandPayload, the mapping tables are combined. Fields marked with a (P) or (P, T) exist only in the respective elements (P)roperty and (T)elemetry. The type field (@type) of a data element can contain a semantic type (e.g., “Temperature”) in addition to the values “Telemetry” and “Property”. In this case, the data type of the element must be a primitive number type [9] for semantically annotated data elements to be transformed into AAS properties. Furthermore, a concept description must be created for the semantic type.

The DTDL component contains a reference to an interface element, a unique name and further optional fields. The AAS Entity element of the proposal from Figure 3 is not suitable for transformation, since it contains a reference to an instance of a sub-twin. On the other hand, the component contains a reference to a model. Consequently, the AAS element ReferenceElement is used for this mapping. The DTDL Command is transformed into an AAS operation, where it should be noted that the field CommandType is obsolete and is therefore only mapped to a qualifier.

Finally, the Relationship element is transformed into the AAS AnnotatedRelationship element. This decision is based on the fields of the DTDL Relationship element, which defines the minimum and maximum number of targets for a relationship, as well as the properties that can be attached to a DTDL relationship to describe it. It should also be noted that the DTDL relationship is a reference to a model and not a concrete instance. Hence, the target of an AAS relationship is always a reference element. Furthermore, the first element of an AAS relationship is a reference to its own submodel and the minimum and maximum number of targets of the relationship is stored in a range element.

For verification, all mapping rules were applied to the 3D printer introduced in Section 2. Listing 1 shows this example for the DTDL interface.
**Listing 1**: Transformation of DTDL interface to AAS submodel.{“qualifiers “: [{“ type”: “@context”,“ valueType”: “string”,“ value”: “ dtmi:dtdl:context;2”,“ modelType”: {“ name “: “Qualifier”}}],“identification”: {“idType”: “Custom”,“id”: “dtmi:com:example:My3DPrinter;1”},“idShort”: “DTDLModel”,“category”: “VARIABLE”,“displayName”: [{“language”: “en”,“text”: “3D−Printer”},{“language”: “de”,“text “: “3D-Drucker”}],“descriptions”: [{“language”: “en”,“text “: “Example model of a 3D printer.”}],“modelType”: {“name”: “Submodel”},“kind”: “Instance”,“submodelElements”: []}

For this purpose, the DTDL model of the 3D printer is divided into different elements and then transformed according to the previous rules. The transformed model was validated by integrating it into a model of the AAS created in the programming language Java.

**Figure 5:**
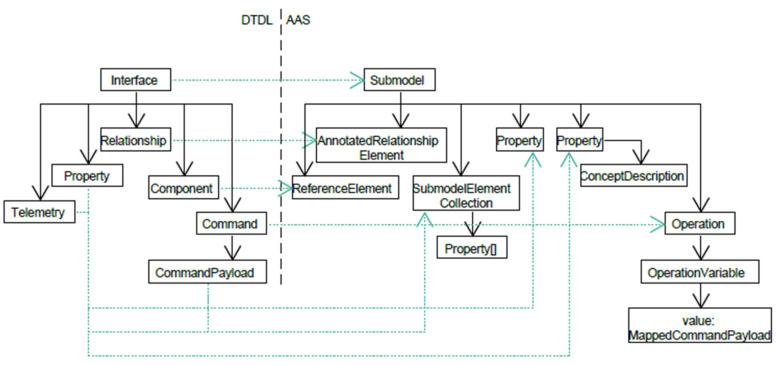
Mapping of the elements, final version.

Each element of the DTDL metamodel was mapped to an element of the AAS. However, some changes were made to the first proposal in Figure 3, which have been updated in the following Figure 5.

## 7. Implementation

This section describes an implementation to transform Digital Twins Definition Language (DTDL) version 2 into AAS version 3.0RC01. For this, the approach defined in Section 5 as well as the classification and mapping rules are used. The chosen programming language for the implementation is Java.

The goal of the implementation is validation of the mapping rules defined in Section 5. To ensure the correct transformation of a DTDL element, the following precondition applies: the DTDL element must conform to the format of DTDL metamodel version 2. This precondition can be checked by validation with the DTDL validator [9]. The result of the transformation is an AAS element, which corresponds to the format of the AAS metamodel version 3.0RC01. Optionally the result can be validated by integrating the transformed element into a reference implementation of the AAS.

**Figure 6:**
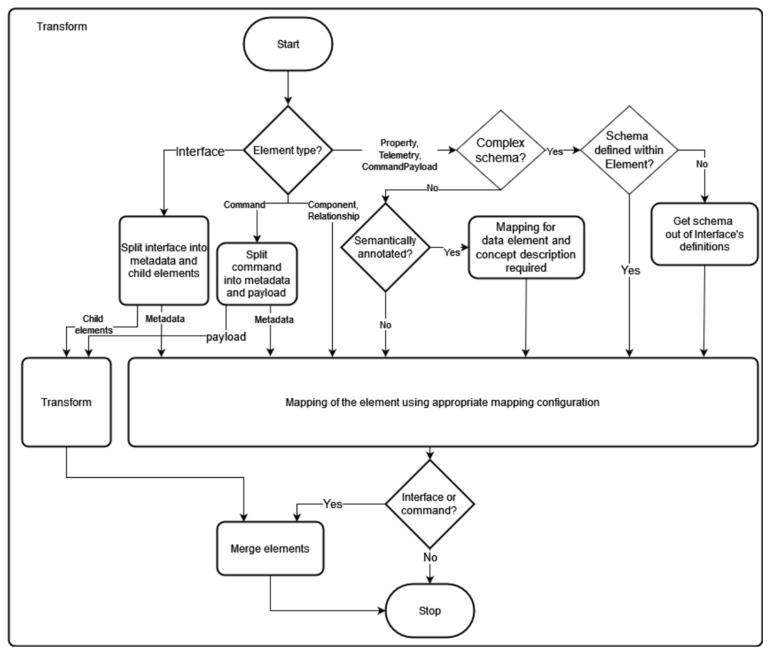
Program flowchart: Selection of the mapping rules.

Figure 6 presents a flow chart in which the decision paths for selecting the correct input are shown. Following a separation of the elements according to the classification from Section 5, any child elements are handled and the correct mapping configuration is loaded. Finally, each input element is transformed using the mapping configuration. In the case of child elements, the same transformation is applied recursively to these elements, and the transformed child elements are inserted into the transformed parent element.

For the mapping of DTDL elements, the mapping configurations are defined in JSON format. They follow the structure of the AAS element into which the transformation shall lead. In addition, there are JSONPath expressions for the fields which are to be described. These expressions describe the position in the source element where the searched value is located. This value is then substituted for the expression. The JSONPath expressions are prefixed with “$” to recognize the JSONPath expressions. For example, the “comment” field of the DTDL component is mapped to an AAS qualifier, which has “comment” as its key and the comment as its value. The JSONPath expression $.[‘comment’] would be evaluated on the JSON element {“comment”: “Hello World”} and generate the output [“Hello World”].

To illustrate the mapping rules, an example of a transformation was carried out on the 3D printer example. Listing 2 shows the DTDL component.
**Listing 2:** DTDL-Component-Element.{“@type”: “Component”,“name”: “filamentStorage”,“schema”: “dtmi:com:example:FilamentStorage;1”,“@id”: “dtmi:com:example:FilamentStorageComponent;1”,“comment”: “A filamentstorage component element”,“displayName”: “FilamentStorage”,“description”: “Filament storage as material source for this printer.”}

Listing 3 shows the transformed output of the program.
**Listing 3:** AAS ReferenceElement.{“value”: {“keys”: [{“type”: “Submodel”,“local”: “false”,“value”: “dtmi:com:example:FilamentStorage;1”,“index”: “0”,“idType”: “Custom”}]},“constraints”: [{“type”: “@id”,“valueType”: “string”,“value”: “dtmi:com:example:FilamentStorageComponent;1”,“modelType”: {“name”: “Qualifier”}},{“type”: “comment”,“valueType”: “string”,“value”: “A filament storage component element”,“modelType”: {“name”: “Qualifier”}}],“idShort”: “filamentStorage”,“category”: “CONSTANT”,“modelType”: {“name”: “ReferenceElement”},“kind”: “Instance”,“displayName”: [{“language”: “en”,“text”: “FilamentStorage”}],“descriptions”: [{“language”: “en”,“text”: “Filament storage as material source for this printer.”}]}

**Figure 7:**
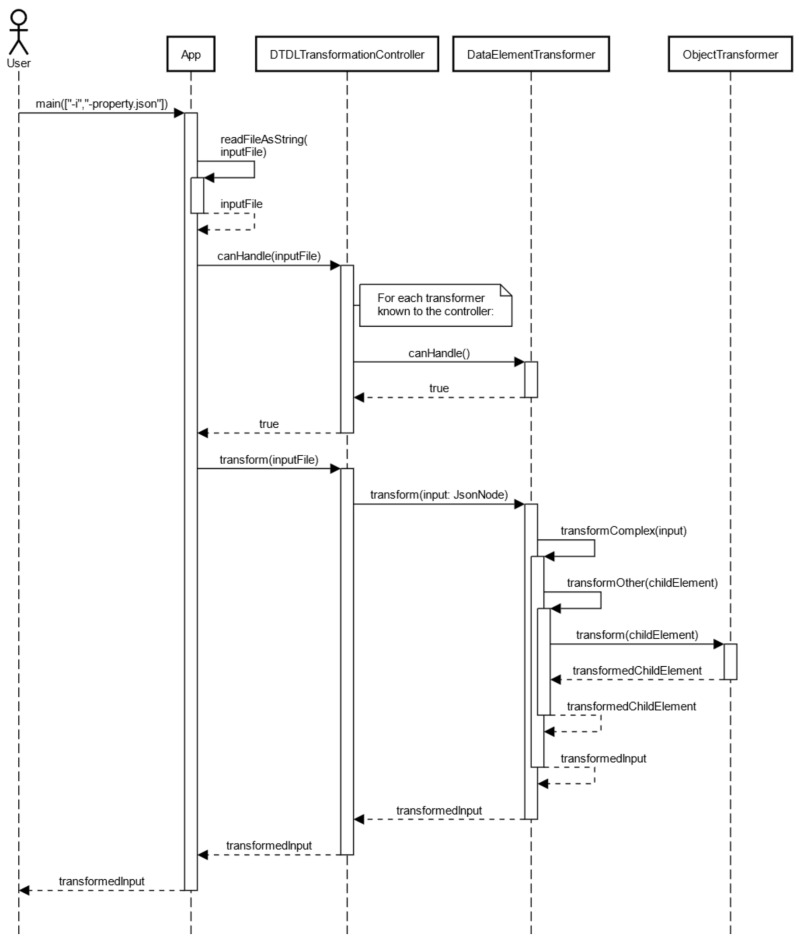
Flow chart of transformation.

The flow chart in Figure 7 outlines the program flow while transforming a DTDL property.

The program developed in this paper transforms the entire DTDL model as well as individual subelements to AAS. The source code of the program is available in [19]. It is worth noting that data elements with a complex datatype array or map cannot be transformed as is, because they cannot be mapped into the AAS using a generic approach. For a given complex type, the code of the program could be extended to define a specific mapping of the complex type into the AAS. The release of AAS model version 3.0 introduced the new element SubmodelElementList, which allows the creation of two-dimensional array lists [20]. This element may present a possible solution for handling maps but was unavailable at the time of implementation. In future work, handling of maps with the new element SubmodelElementList should be attempted. In addition, although there is a mapping rule for concept descriptions, the current state of the program cannot output them, resulting in semantically annotated data elements being transformed without an associated concept description. This is a structural problem that can be solved, for example, with a separate output of the concept description but requires significant time investment to implement in the program.

The extension and structural change in the transformation via the mapping configurations is not possible without also making changes to the program code. Subsequently, these changes to the metamodel of DTDL and AAS would result in a change to the program code. A more complex program could load mapping rules from a mapping file to prevent program code changes if mapping rules need to be changed.

Furthermore, a reverse transformation of the transformed AAS into the DTDL is currently not implemented. The approach of Mayrbäurl et al. [3] cannot be used for the reverse transformation since all elements of the AAS are mapped to variations of the DTDL interface element resulting in two successive transformations not producing the same element. Further work in this area could result in the development of a more general approach to a transformation or a feasibility analysis of a transformation in the other direction. However, real usage scenarios in which one model is usually transformed into another without reverse transformations are not limited by this. A practical example consists of an IoT device with a DTDL model that is sold to a customer. This customer chooses to operate the device digital twin with AAS models and tools instead of DTDL. With the implemented program, an AAS output file can be generated from the DTDL model. If the model did not include complex types or maps, then the output file can be used as is. Otherwise, the customer must transform the complex types and maps manually into the AAS.

The transformation of DTDL to AAS was tested with several DTDL input files and resulted in complete machine-readable AAS files. Since the transformation is a generic, repeatable process, the quality of the output model is dependent on the quality of the input.

## 8. Summary and Conclusions

This paper demonstrated that each element of the DTDL metamodel can be mapped to an element of the AAS. The chosen approach is inspired by the work of Pakala et al. [2] since there are structural similarities between the DTDL metamodel and that of the Thing Description. The classification of the DTDL metamodel elements has contributed to a clearer proposal of a transformation by unifying the mapping rules. In addition, the examples of transformations provided in the previous section have demonstrated that the defined rules produce valid AAS elements. Limitations currently exist in the form of mapping elements with complex types and maps into the AAS.

The topic of digital twins is still evolving, and various standards have emerged to address different use cases and domains. Each of these standards provides a common language and structure for communicating information about the physical assets and their digital representations. The choice of a digital twin standard depends on the domain, requirements and context in which it will be applied. To realize the full potential of digital twins and to ensure interoperability, mapping between different digital twin standards is essential. Additionally, the entities specifying digital twin standards should analyze other relevant standards to enable easier mappings.

In this paper, the relevant standards and specifications from a structural point of view were analyzed and the possibility of their transformation into the AAS specification was examined. To perform the analysis, a common example was used to model DTs in all considered DT standards. Based on the requirements, the DTDL standard was selected as the most feasible for transformation into the AAS.

Additionally, a concept for mapping DTDL to AAS at the metamodel level was proposed and a solution to transform a DTDL-compliant DT into an AAS-compliant DT was developed. This will enable the provisioning of a DTDL entity in the form of an AAS so that a DTDL DT can be used with AAS tools and thus support AAS use cases.

The proposed solution can enable seamless communication and integration between DTs using these two standards and will facilitate the exchange of data across different systems, e.g., applications based on Microsoft Azure Digital Twin and the Industry 4.0 domain. The next step will include the extension of the proposed approach to ensure bidirectional mapping. Furthermore, DTDL and AAS are evolving standards whose specifications may be extended or changed, requiring the mapping to also be updated in the program code.

## Figures and Tables

**Table 1:** Extract of requirements for modeling digital twins [6].

Requirements
Digital twins use models to represent aspects of their assets in a structured way. The digital twin must support the entire product lifecycle of its asset, from conception, through design and development, to its deployment and maintenance. Portability and reusability, as well as scalability, must be provided. Instances of digital twins must be able to interact. Integration/composition of digital twins (digital sub-twins) must be supported. Interchangeability of digital twin instances of the same model must be supported. (An instance is a digital twin of the model already in use). A standardized definition of the structure and basic capabilities (building blocks) of a digital twin must be provided. A digital twin must be accompanied by a standard definition of its term and a taxonomy.

**Table 2:** AAS—Compliance with the requirements.

Requirement	Fulfillment	Comment
Models	Yes	Metamodel of the AAS is defined in [8].
2. Product life cycle	Yes	Fulfilled by the concept of templates, as this allows an AAS model to be developed at the planning stage.
3. Portability, reusability, scalability	Yes	Portability: Through the .aasx format, as well as various serialization options (e.g., JSON); Reusability: Given by the concept of templates; Scalability: By dividing an AAS into submodels, the structure of a twin can be scaled as desired.
4. Interaction	Yes	Fulfilled by the ability to reference external AASs.
5. Composition	Partially	Composition and integration of AASs in a single model is not possible because an AAS represents only one asset [8]. However, sub-twins can be modeled by entities.
6. Interchangeability of instances	Yes	Supported through standardization via submodel templates. Submodels of different AASs can be exchanged if they use the same template.
7. Standardized definition, capabilities	Yes	Standardized definition: [8]; Capabilities: functions, events, properties can be modeled [5]
8. Definition DT, Taxonomy in model	Yes	Defined by the AAS metamodel [8].

**Table 3:** DTDL—Compliance with the requirements.

Requirement	Fulfillment	Comment
Models	Yes	Specification for DTDL models under [9].
2. Product life cycle	Yes	Design of a digital twin is possible through DTDL models. The implementation of the model allows usage and maintenance of the asset, as the basic functionality of a DT is provided.
3. Portability, reusability, scalability	Yes	Portability: DTDL is based on JSON-LD and models are created in JSON format; Reusability: a DTDL model can be reused for different assets with equivalent capabilities; Scalability: Scalability is provided by relationships between and composition of digital twins in DTDL format.
4. Interaction	Yes	Supported through relationships between DTDL models.
5. Composition	Yes	Supported through components within an interface element.
6. Interchangeability of instances	No	Instances of DTDL models do not always implement the same elements (see minMultiplicity/maxMultiplicity of relationships in [9]).
7. Standardized definition, capabilities	Yes	Standardized definition: [9], Capabilities: functions, events, properties can be modeled [5].
8. Definition DT, Taxonomy in model	Yes	Assets described by DTDL are “Plug & Play devices, device digital twins, and logical digital twins” [9]. Terms of the elements of a DTDL twin are defined in [9].

**Table 4:** NGSI-LD—Compliance with the requirements.

Requirement	Fulfillment	Comment
Models	Yes	Specification for NGSI-LD models [10].
2. Product life cycle	Yes	Ontologies provide modeling for specific application areas (design), which can be used for the application of digital twins.
3. Portability, reusability, scalability	Yes	Portability: NGSI-LD is based on JSON-LD; Reusability: One model can be used for any number of assets; Scalability: Using relation-elements, larger systems can be modeled.
4. Interaction	Yes	Supported through relationships in the NGSI-LD metamodel.
5. Composition	Partially	Sub-twins can be modeled via relationships, i.e., not directly within the model of the twin.
6. Interchangeability of instances	Yes	Models define all the properties and relationships that a twin possesses.
7. Standardized definition, capabilities	Yes	Standardized definition: [10], Capabilities: Properties can be modeled, events only by subscribing to properties. Functions cannot be modeled [5].
8. Definition DT, Taxonomy in model	Partially	A concrete definition of the term “digital twin” is not given in the specification. The terms of the elements of a NGSI-LD twin are defined in [10].

**Table 5:** Vorto—Compliance with the requirements.

Requirement	Fulfillment	Comment
Models	Yes	Vorto metamodel.
2. Product life cycle	Yes	Design and development can be supported by creating a Vorto model of the asset. Deployment and maintenance of the asset are supported using the model.
3. Portability, reusability, scalability	Partially	Portability: Serialization into different formats is possible [11]; Reusability: Vorto models could be published until 2021 in an Eclipse Vorto repository [11]; Scalability: Representation of relationships and composition of twins is not possible.
4. Interaction	No	Relationships and compositions of twins are not defined in the metamodel [11].
5. Composition	No	See 4.
6. Interchangeability of instances	Yes	Model instances implement the same number of function blocks. In addition, the prefix mandatory before fields ensures that an asset must have necessary properties [11].
7. Standardized definition, capabilities	Yes	Standardized definition: [11], Capabilities: functions, events and properties can be modeled
8. Definition DT, Taxonomy in model	Yes	The term “digital twin” was defined in the specification of Vortolang in [11]. The taxonomy of the metamodel is also defined in [11].

**Table 6:** WoT—Compliance with the requirements.

Requirement	Fulfillment	Comment
Models	Yes	The specification is published in [14].
2. Product life cycle	(No)	The creation of templates in the form of Thing Models is supported in version 1.1, but this is only available in draft form [13].
3. Portability, reusability, scalability	Partially	Portability: Models are available in JSON format; Reusability: See 2. Through templates, TD models can be used for different twins; Scalability: Composition of twins is not explicitly defined in the TD metamodel, but relationships between twins can be modeled.
4. Interaction	Yes	Supported through the representation of relationships between twins.
5. Composition	No	This functionality is not explicitly defined in the TD.
6. Interchangeability of instances	(No)	A unification of twins is achieved by the introduction of Thing Models in version 1.1 [13].
7. Standardized definition, capabilities	Yes	The structure is defined in [14] and basic capabilities according to [5] are available.
8. Definition DT, Taxonomy in model	Yes	The term “Thing”, as well as the taxonomy of the metamodel, are defined in [14].

**Table 7:** Comparison of the standards and specifications with the AAS.

Capability	AAS	DTDL	NGSI-LD	Vorto	WoT TD
Telemetry data	✓	✓	✓	✓	✓
Functions	✓	✓	X	✓	✓
Configuration	✓	✓	✓	✓	✓
Identifiers	0	-	-	-	-
Relations	✓	✓	✓	X	✓
Composition	✓	✓	X	X	✓
Semantic annotation	✓	(✓)	✓	✓	✓
Events	✓	X	X	✓	✓
Time-series data	X	X	✓	X	X
Geo data	X	✓	✓	X	X
Asset connection	X	X	X	X	✓
Security definitions	(✓)	X	X	X	✓

**Table 8:** Mapping of the DTDL interface element.

Field of the Data Element: Data Type	Submodel Field
@id: DTMI @type = “Interface” @context = “dtmi:dtdl:context;2” comment: String contents description: String displayName: String extends: Interfaces schemas: Interface Schemas	Identifier modelType = “Submodel” qualifier qualifier SubmodelElements Description displayName semanticId -

## Data Availability

Not applicable.
